# Effectiveness of Mobilization with Movement on the Management of Knee Osteoarthritis: A Systematic Review of Randomized Controlled Trials

**DOI:** 10.1155/2021/8815682

**Published:** 2021-05-03

**Authors:** Gidey Gomera Weleslassie, Melaku Hailu Temesgen, Abayneh Alamer, Gebrerufael Solomon Tsegay, Teklehaimanot Tekle Hailemariam, Haimanot Melese

**Affiliations:** Department of Physiotherapy, School of Medicine, College of Health Sciences and Ayder Comprehensive Specialized Hospital, Mekelle University, Mekelle, Ethiopia

## Abstract

**Background:**

Osteoarthritis is becoming a global major cause of pain and functional disability worldwide, especially in the elderly population. Nowadays, evidence shows that mobilization with movement (MWM) has a beneficial effect on knee osteoarthritis subjects. However, its adequacy remains unclear.

**Objective:**

To review the best available evidence for the effectiveness of MWMs on pain reduction and functional improvement in patients with knee osteoarthritis.

**Methods:**

A comprehensive search of literature was conducted using the following electronic databases: Google Scholar, PubMed, Physiotherapy Evidence Database (PEDro), Science Direct, Cochrane Library, and Scopus. Only randomized controlled trials (RCTs) were included, and the methodological quality of the studies was appraised using the PEDro scale. It was reported according to the guideline of the PRISMA statement.

**Results:**

A total of 15 RCTs having 704 participants were included. The present systematic review suggests that there were significant differences between MWM groups and control groups in terms of visual analogue scale (VAS), Western Ontario and MacMaster Universities Osteoarthritis Index (WOMAC) scale, and flexion range of motion.

**Conclusions:**

This systematic review demonstrated that MWM was effective to improve pain, range of motion, and functional activities in subjects with knee osteoarthritis.

## 1. Introduction

Osteoarthritis (OA) is a chronic degenerative disease characterized by the deterioration of the cartilage in the joints, creating stiffness, pain, and impaired movement [1, 2]. Osteoarthritis a leading cause of pain and functional disability in both developed and developing countries, especially in the elderly population [3, 4].

The knee joint is the most commonly affected joint by OA due to its weight-bearing requirement, high mobility, and lack of intrinsic stability [5]. It leads to limitations in activities of daily life and impairment in the quality of life because of the accompanying pain and morning stiffness in the joint [6].

According to the World Health Organization (WHO), OA affects 9.6% of men and 18% of women over 60 years of age [7]. Generally, 43.4 million people in the world were affected by OA associated with a disability by the year 2020 [8, 9]. Moreover, there is an increasing need for urgent attention to this disease because of the societal trends in the population such as aging, obesity prevalence, and joint injury, estimating that the number of people affected by OA will increase by about 50% over the next 20 years [10, 11].

The management of knee OA needs a multidisciplinary approach. The conservative treatment forms for knee OA comprise pharmacological and nonpharmacological modalities. Nonsteroidal anti-inﬂammatory drugs are mostly used for pain relief and stiffness caused by OA with their numerous side effects, particularly on the gastrointestinal tract, making the treatment unsustainable [12, 13], whereas the nonpharmacological treatments such as manual therapy, weight management strategies, kinesiotherapy, resistance strengthening exercise, aerobic conditioning, and physical agent modalities such as electrotherapy were used to relieve pain, to reduce or eliminate complications, and to prevent disease progression for knee OA [14–21].

Mobilization with movement (MWM) is a manual therapy technique that is used most frequently for the management of musculoskeletal conditions [22]. It was initially advocated by Brian Mulligan and has been proposed as a novel manual therapy technique to treat a variety of upper and lower limb joint-related soft tissue conditions [23, 24]. In this technique, the physiological movement is performed in a pain-free manner [24] with accessory glides being applied in the direction towards the opposite of the previously painful movement to have the greatest improvement [25].

MWMs has shown promising various therapeutic beneﬁts such as reduction of pain and improved range of motion [23, 26]. The rationale for the use of MWM techniques is directed towards correcting positional faults at the joint [27].

Previous evidence has furnished the beneficial effects of MWM on different peripheral joints [23, 25, 28]. Nowadays, studies mentioned that MWM had a beneficial effect on knee OA patients. Nevertheless, its adequacy remains unclear. Therefore, the aim of this review was to analyze the effectiveness of MWM on pain reduction and functional improvement in patients with knee OA.

## 2. Methods

### 2.1. Design and Protocol Registration

This systematic review was conducted in accordance with the Preferred Reporting Items for Systematic Reviews and Meta-Analyses (PRISMA) guideline [29] with International Prospective Register of Systematic Reviews (PROSPERO) registration number CRD/42020193092.

### 2.2. Search Strategy

An extensive literature search was performed to identify all eligible randomized controlled trials from inception to September 2020. Systematic and comprehensive searches were conducted in electronic databases such as Google Scholar, Physiotherapy Evidence Database (PEDro), Scopus, Science Direct, Cochrane Library, and PubMed. The search was made using the following keywords: mobilization with movement, knee osteoarthritis, and randomized controlled trial. As the topic titles speckled among the databases, various combinations of the keywords were used: “Mulligan's mobilization/mobilization with movement/sham/placebo mobilization with movement” and “osteoarthritis/knee joint pain/Arthritis/degenerative knee arthritis” and “randomized controlled trial.”

### 2.3. Eligibility Criteria

Studies for this review were assessed according to the following eligibility criteria.

#### 2.3.1. Type of Studies

Only randomized controlled trials (RCTs) published in English and full-text availability.

#### 2.3.2. Participants

Studies were included if they recruited male and/or female, diagnosed with knee OA, and subjects above 40 years of age.

#### 2.3.3. Interventions

Intervention groups received MWM and MWM combined with conventional therapy (usual care and exercise) for the treatment of knee OA.

#### 2.3.4. Comparisons

The control group received sham/placebo MWM, exercise, usual care, McConnell patella taping, and Maitland mobilization.

#### 2.3.5. Primary Outcome Measures

The Visual analog scale (VAS), numeric pain rating scale (NPRS), McMaster Universities Arthritis Index (WOMAC) scale, Time Up and Go test (TUG), and range of motion (ROM).

#### 2.3.6. Secondary Outcome Measures

A six-minute walk test, pain-free squat angle, Aggregated Locomotor Function (ALF), and Knee injury and osteoarthritis outcome scale (KOOS).

### 2.4. Exclusion Criteria

The exclusion criteria established for this review were observational studies, quasiexperimental studies, studies not having full access, and RCTs published in other than English languages, and results obtained from theses/dissertations, conference proceedings, abstracts, and websites were excluded from this review.

### 2.5. Study Selection

The study selection process was performed by four reviewers (G. G, M. H, T. T, and H. M). Only randomized control trials and studies intervening knee OA with MWM were included in this review. Any disagreement between the reviewers should be consulted by two reviewers (G. S and A. A) to reach a consensus.

### 2.6. Hierarchy of Evidence

Four reviewers independently assessed all sources of the papers, and the level of each study was determined according to the hierarchical system of Lloyd-Smith. The level of evidence reveals the degree to which bias has been considered within study design, with a lower rating on the hierarchy indicating less bias. Merely studies that scored between 1b and 2a on the Lloyd-Smith scale were included in this systematic review. In this approach, we could ensure that MWM for knee OA supported by this review was based on results of high-level evidence.

### 2.7. Data Extraction

Based on a predetermined extraction tool, three authors (G.G, A.A, and G.S) independently extracted relevant data from each article. The following data were extracted from each trial: general study information (title, authors' name, year of publication, and country of study), OA deﬁnition (severity measure, type, and duration), number of participants in the treatment and control group, mean follow-up time, type of treatment, mean age of the participants, inclusion and exclusion criteria of the participants, primary outcome measures, study design, study findings, and conclusions.

### 2.8. Risk-of-Bias Assessment

The qualities of the eligible studies were assessed using the Physiotherapy Evidence Database (PEDro) Scale [30]. The PEDro scale includes 11 items, in which the first item assesses the external validity and the remaining 10 items assess the internal validity, examining random allocation, concealment of allocation, baseline equivalence, blinding procedure, ‘intention to treat' analysis, adequacy of follow-up, between-group statistical analysis, and measurement of data variability. This review considered trials with a score of 5 to 7 as moderate quality and a score of ≥8 as a high-quality study (Table 1).

## 3. Results

### 3.1. Literature Search

A total of 1198 articles were identified by the searching strategy. After adjusting for duplicates, 768 remained. After the title and abstract screening of studies, 365 studies were expelled. Subsequently, by full content screening out of 38 articles, 15 randomized controlled trials were included in this review (1).

### 3.2. Description of the Studies

The characteristics of the included studies are illustrated in Table 2. The included studies were published between 2010 and September 2020. Overall, 704 participants with knee OA aged from 40 to 70 years were included. The average age of the participants ranged from 47.47 ± (0.61) to 58.5 ± (4.36) in the experimental group [38, 44] and 47.47 ± (0.61) to 59.4 ± (6.57) [38, 44] in the control group. The follow-up duration of the experimental and control group ranged from two days to twelve months [36, 40], with the majority of the studies having a follow-up duration of around two to three weeks.

The mean PEDro score of the studies was 6.7 (range: 5–9) (Table 3). Two trials [36, 44] scored 8, and four trials [5, 35, 39, 40] scored 9 on the PEDro scale, which was the highest possible score given the intervention, as it would not be feasible to blind clinicians.

### 3.3. Risk of Bias within the Studies

The methodological qualities of the included studies are summarized and reported in Table 3. Out of the 15 randomized controlled trials, 9 articles reported about the procedure of proper randomization sequence and six randomized controlled trials conducted by Shenouda [31], Gupta and Heggannavar [33], Kulkarni and Kamat [34], Kiran et al. [38], Saddam Hussain Shaik et al. [41], and Pawar et al. [42] had not stated the randomization method. Six trials reported concealed allocation, and the majority of the articles had not clearly reported a concealed allocation method. In study performance bias, merely 3 of the randomized controlled trials were found to be double-blinded and the other remaining articles are single-blinded. Six randomized controlled trials conducted by Lalnunpuii et al. [5], Rao et al. [35], Kaya Mutlu et al. [36], Bhagat et al. [39], Alkhawajah and Alshami [40], and Nigam et al. [44] had a blinded outcome assessor.

### 3.4. Interventions

Randomized control trials comparing the effectiveness of MWM, MWM combined with conventional physiotherapy and comparison/control group: sham/placebo MWM, and/or conventional physiotherapy, usual care, myofascial release, KT, exercise, McConnell patella taping, and Maitland mobilization intervention were included.

### 3.5. Outcome Measures

The outcome measures for each of the fifteen trials are presented in Table 2 All the studies included outcome measures for pain and functional disability status. The pain intensity was measured by the Visual Analogue Scale (VAS) in eleven studies [5, 31–34, 36–38, 40, 42, 44], Numerical Pain Rating Scale (NPRS) in three studies [35, 39, 43], and KOOS in one study [41]. Functional disability status was measured by the Western Ontario and McMaster Universities Osteoarthritis Index (WOMAC) in ten studies [5, 31–33, 36–38, 40, 43, 44], Time Up and Go in three studies [35, 39, 44], and 6-minute walk test in one trial [34] for subjects with knee OA.

### 3.6. Effects of MWM on Pain Reduction

The effects of MWM intervention in subjects with knee OA are summarized in Table 2. Out of the 15 included trials, 14 of them reported that knee pain was significantly improved in the MWM groups compared to the control group [5, 31, 33–44]. Only one study reported that the MWM group had no improvement in knee pain compared to the control groups [32].

### 3.7. Effects of MWM on Knee Joint ROM

From the total included trials, nine of them had assessed knee ROM. Out of these, eight trials reported that MWM has positive effects on joint range of motion for OA patients compared to the control groups [5, 31, 33, 36, 38, 40, 43, 44]. Conversely, only one study reported that the MWM group had no improvement of knee ROM compared to the control group [32].

### 3.8. Effects of MWM on Functional Status

Out of the included trials, fourteen of them had assessed functional status. Out of these trials, thirteen of them had reported that MWM has positive effects on functional activities in OA patients compared to the control groups [5, 31, 33–39, 41, 43, 44]. However, two studies reported that the MWM group had no significant effect on knee functional status in patients with knee OA [32, 40].

## 4. Discussion

This review of RCTs has been designed to investigate the effectiveness of MWM in subjects with knee OA. To the extent of our knowledge, this is the preliminary review to systematically evaluate the effectiveness of MWM among subjects with knee OA. In this review, 15 recent RCTs were included, which investigated the effectiveness of MWM in subjects with knee OA as compared with control interventions.

Most of the included studies published that MWMs is effective in improving pain, range of motion, and physical functioning in patients with knee OA. Most of the studies used the same outcome measure, particularly VAS, WOMAC, and knee ROM, for pain and functional impairment. Ten of the trials had assessed pain using a VAS [5, 31–34, 36–38, 40, 44], three trials used the NPRS [35, 39, 43], and the Knee injury and Osteoarthritis Outcome Scores (KOOS) pain subscale in one study [41] suggested that MWM had positive effects for subjects with knee OA on pain reduction. The results in this review are consistent with the previous systematic reviews on peripheral joints reporting positive clinical effects of MWM [23, 25, 28].

Shenouda [31] reported that MWM had positive effects for subjects with knee OA on pain reduction and functional disability. Besides, MWM has no statistically significant difference in the improvement of knee ROM in both the interventional and control groups. However, in within- and between-group analysis of pre- and posttreatment, there was statistical significance in all outcome measures. In contrast, a study conducted by Kandada andHeggannavar [32] showed that the intergroup analysis shows an insignificant difference in all the outcome measures. But, the intragroup comparison shows a significant difference in pain reduction, functional improvement, and knee ROM.

Another study investigated by Gupta et al. [33] showed that MWM had significant improvement in pain reduction, functional disability, and knee joint proprioception in OA knee participants. This could mean MWM may have had beneficial effects on joint nutrition because of the squeezing out of the fluid during each compression and imbibing of fluid when the compression is removed [45]. Normally, squeezing occurs when the mobilization technique is performed and imbibing of fluid occurs when the joint is relaxed. This could possibly be the reason for a reduction in pain and a subsequent improvement in ROM and function that was found at the end of the treatment session. Likewise, Lalnunpuii et al. [5] reported more significant relieving pain, increasing ROM, and functional capacity were found in the intervention group compared to the control group in females with knee OA. This could be beacuse MWM might provide a stretching effect on the joint capsules and muscles, thus restoring normal arthrokinematics or decreasing pain by stimulation of joint mechanoreceptors, which consequently inhibits nociceptive stimuli and improved motor control [46, 47]. Kulkarni and Kamat [34] showed that significant reductions in pain (*P* < 0.05) and improvement in 6-minute walk test distance covered during posttreatment sessions in both groups. However, posttreatment distance covered in the experimental group (mean = 37, SD = 16.882) was greater than that in the control group (mean = 35, SD = 23.146). Besides, Rao et al. [35] reported that both groups have shown a significant effect in reducing pain and improving functional mobility in subjects with knee OA immediately after treatment. Similarly, the study by Kaya Mutlu et al. [36] showed that MWM is superior in reducing pain, improving quadriceps muscle strength, knee range of motion, and functional level than the control group in knee OA participants. This could be due to the repeated motion of MWM, which might alter the concentrations of anti-inflammatory mediators in the joint, which might consequently inhibit nociceptors [48]. Another possible reason could be due to psychological effects such as a reduction in fear avoidance associated with movement [49].

Varma and Purohit [37] found that MWM combined with conventional exercise groups showed better significant improvement in reducing pain, improving function than the conventional exercise group after 2 weeks of intervention. In the intervention group, the improvements could be because of biomechanical and neurophysiological mechanisms of MWM that may produce pain at the spinal level (pain gate mechanisms) [50]. Besides, a study by Kiran et al. [38] found that both groups showed a significant effect on the improvement of pain, knee range of motion, and functional ability after two weeks of intervention in patients with knee OA.

Bhagat et al. [39] investigated that MWM produced direct effects in reducing knee pain and improving functional mobility in knee OA as compared with the placebo group. This could be because of the biomechanical mechanisms in Mulligan's MWM concept [51, 52]. The correction of positional faults by the treatment glides used in the intervention group could have quickly repaired the normal kinematics of the osteoarthritic knee producing instant pain relief. Furthermore, a study conducted by Alkhawajah and Alshami [40] suggested that MWM had a superior effect in reducing pain, improving physical function (TUG), and knee flexion than the sham group. However, WOMAC (*P*=0.067) has no significant effect on patients with knee OA. This could be because the grade of OA was relatively low, which may denote a nonmajor limitation of functional activity and the duration of the follow-up was short (2 days) which might not be sufficient for a perceived improvement in daily activities.

Another study conducted by Saddam Hussain Shaik et al. [41] reported that MWM had a significant improvement in pain reduction and quadriceps peak torque compared to the control group. In the intervention group for pain, the reduction may be because of the inspiration that MWM sedates an agitated, expedited system, significantly the dorsal horn, by offending it with painless normality it is been freckled to receive. Another attainable reason may be central mechanisms area unit concerned as there is activation of the nonopioid mediate drizzling pain restrictive system [25, 53]. In addition, the mechanism responsible for the improvement of quadriceps peak torque is believed to be the arthrokinetic reflex, defined as the influence of joint mechanoreceptor afferents on muscles around the joint [54]. Pawar et al. studied the effect of MWM in subjects with knee OA. A randomized control trial shows that the MWM group showed greater improvement when compared with the control group on pain [42].

Shamim et al. [43] investigated that reduction of pain and ROM showed a significant effect in the MWM group compared to the control group. In the study conducted by Nigam et al. [44] MWM combined with usual care suggested that there is a significant effect of MWM in favor of the intervention group for WOMAC and VAS compared to the control group. The superior pain reduction in the experimental group could be because MWM may decrease nociceptive inputs while increasing nonnociceptive inputs via activation of peripheral mechanoreceptors [53]. However, there were no significant differences between groups for functional mobility as measured with the TUG immediately after the intervention. The improvement seen in both groups could be due to the positive effects of exercise. Exercise reduces pain, increases muscle strength, and improves control around the affected joint [55].

### 4.1. Limitations

This systematic review has some limitations. The first limitation of this review is that a language bias is possible as only those studies that were available as full text in English were included. The second limitation of this review is that the outcome measures were not similar across the RCTs included in this review. A meta-analysis was not conducted because of the heterogeneous nature of the studies, and this could have been valuable for the effect of mobilization with movement. Third, studies with short follow-up duration were included because a longer treatment duration could likely result in a significant intergroup difference. Lastly, only one of the available studies scrutinized the long-term effects of the MWMs on knee OA [44]. Based on the systematic appraisal of the current literature, any future research studies should consider the limitations of the previous studies in order to improve the quality of evidence in this field.

### 4.2. Clinical Implication

This review suggests that MWM appears to reduce pain, improves knee range of motion, and improve physical functioning in subjects with knee osteoarthritis.

## 5. Conclusions

The findings of this systematic review suggest that Mulligan's MWM could be a treatment option among subjects with knee osteoarthritis. This review supports the evidence that Mulligan's MWM reduces pain, improves knee range of motion, and physical functioning of subjects with knee osteoarthritis.

## Figures and Tables

**Figure 1:**
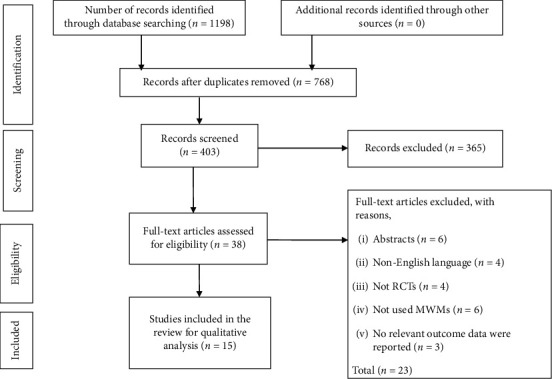
PRISMA flowchart of the study.

**Table 1:** Quality assessment of controlled intervention studies.

PEDro scale items	Shenouda (2013) [31]	Kandada and Heggannavar (2015) [32]	Gupta and Heggannavar (2015) [33]	Lalnunpuii et al. (2017) [5]	Kukarni and Kamat (2017) [34]	Rao et al. (2018) [35]	Kaya Mutlu et al. (2018) [36]	Varma and Purohit (2020) [37]	Kiran et al. (2018) [38]	Bhagat et al. (2019) [39]	Alkhawajah and Alshami (2019) [40]	Saddam Hussain Shaik et al. (2019) [41]	Pawar et al. (2019) [42]	Mahmooda et al. (2020) [43]	Nigam et al. (2020) [44]
Eligibility	Yes	Yes	Yes	Yes	Yes	Yes	Yes	Yes	Yes	Yes	Yes	Yes	Yes	Yes	Yes
Random allocation	Yes	Yes	Yes	Yes	Yes	Yes	Yes	Yes	Yes	Yes	Yes	Yes	Yes	Yes	Yes
Concealed allocation	No	No	No	Yes	No	Yes	Yes	No	No	Yes	Yes	No	No	No	Yes
Baseline comparability	Yes	Yes	Yes	Yes	Yes	Yes	Yes	No	Yes	Yes	Yes	Yes	No	Yes	Yes
Blind participants	No	No	No	Yes	No	Yes	Yes	No	No	Yes	Yes	No	Yes	No	Yes
Blind therapist	No	No	No	No	No	No	No	No	No	No	No	No	No	No	No
Blind assessor	No	No	No	Yes	No	Yes	Yes	No	No	Yes	Yes	No	No	No	Yes
Adequate follow-up	Yes	No	Yes	Yes	Yes	Yes	Yes	Yes	Yes	Yes	Yes	Yes	Yes	Yes	Yes
Intention: to treat analysis	No	Yes	No	Yes	Yes	Yes	No	Yes	No	Yes	Yes	Yes	No	Yes	Yes
Between-group comparison	Yes	Yes	Yes	Yes	Yes	Yes	Yes	Yes	Yes	Yes	Yes	Yes	Yes	Yes	Yes
Point estimate and variability	Yes	Yes	Yes	Yes	Yes	Yes	Yes	Yes	Yes	Yes	Yes	Yes	Yes	Yes	Yes
Total score	5/10	5/10	6/10	9/10	6/10	9/10	8/10	5/10	5/10	9/10	9/10	6/10	5/10	6/10	8/10
Graded approach	Moderate	Moderate	Moderate	High	Moderate	High	High	Moderate	Moderate	High	High	Moderate	Moderate	Moderate	High

**Table 2:** Summary of included randomized control trials.

Authors (year)	Patient characteristics, sample size, and mean age	Intervention	Frequency, follow-up time	Outcome measure	Results	Conclusion
Shenouda (2013) [31]	Source : 45outpatients (GA = 15, GB = 15, GC = 15) and mean age (S. D) : GA = 51.93 ± 6.51, GB = 52.2 ± 5.44, and GC = 50.07 ± 5.73	GA = MWM plus exercise GB = SWT plus exercise GC = only exercise	Thrice per week for 4 weeks	VAS WOMAC ROM	Significant difference in pain and functional disability was found in GA and GC. But, no significance difference was found between GA and GC for ROM	MWM was effective in relieving knee pain and functional disability

Kandada and Heggannavar (2015) [32]	Source : 64 outpatients (GA = 32, GB = 32) and mean age (S. D) : GA = 50.13 ± 6.94 and GB = 54.72 ± 6.25	GA = MWM plus CPT GB = MIMG protocol plus CPT	2 weeks	VAS ROM WOMAC	Significant intragroup (*P* < 0.001) difference was found. But, intergroup comparison is not significant in all variables	Both MWM and MIMG protocol are effective in treating OA knee

Gupta and Heggannavar (2015) [33]	Source : 60 outpatients (GA = 20, GB = 20, GC = 20) and mean age (S. D) : GA = 54.10 ± 6.69, GB = 50.95 ± 5.97, and GC = 53.35 ± 6.34	GA = MWM plus CPT GB = proprioceptive exercise plus CPT GC = proprioceptive exercise plus MWM plus CPT	3 sets of 10 repetitions, 1 session per day for 2 weeks	VAS WOMAC ROM	There were statistically significant changes in all outcome measures of GA and GC	Statistically significant improvement was noted in knee joint proprioception on OA knee participants with Mulligan's MWM

Lalnunpuii et al. (2017) [5]	Source : 45 outpatients (GA = 15, GB = 15, GC = 15) and mean age (S. D) : GA = 49.46 ± 5.48, GB = 48.46 ± 6.86, and GC = 47.93 ± 5.61	GA = MWM plus exercise GB = Maitland mobilization plus exercise GC = exercise only	Thrice per week for 4 weeks	VAS ROM WOMAC	All outcome parameters (*p* < 0.05) are statistically improved in the experimental group compared with the control group	MWM is more effective than Maitland mobilization in relieving pain and increasing ROM and functional capacity in females with knee OA

Kulkarni and Kamat (2017) [34]	Source : 30 outpatients (GA = 15, GB = 15) and mean age: not stated	GA = MWM plus CPT GB = CPT	One session per day for 3 days	VAS 6-minute walk test	Statistically significant (*p* < 0.05) reduction in VAS and marked improvement in the distance covered in the experimental group	MWM was effective in reducing pain, and showed marked improvement in the 6-minute walk test in the experimental group

Rao et al. (2018) [35]	Source : 30 outpatients (GA = 15, GB = 15) and mean age 51.2 ± 9.2	GA = MWM followed by Maitland mobilization GB = Maitland mobilization followed by MWM	1–3 oscillations per second, 3 repetitions, for three days	NPRS TUG Pain-free squat angle	Within intervention, both groups showed significant changes (*p* < 0.001) in all outcome measures	Both are equally effective in reducing pain and improving functional mobility and pain-free squat angle for knee OA

Kaya Mutlu et al. (2018) [36]	Source : 72 outpatients (GA = 24, GB = 24, G = 24) and mean age (S. D): GA = 54.19 ± 7.34, GB = 56.29 ± 6.64, and GC = 57.77 ± 6.24	GA = MWM plus exercise GB = PJM plus exercise GC = electrotherapy plus exercise	Thrice per week at 1-year follow-up	WOMAC VAS ROM ALF	WOMAC, VAS, and knee ROM are significantly improved in the experimental group compared to the control group	MWM and PJM were superior to the control group in pain, knee ROM, quadriceps muscle strength, and functional level

Varma and Purohit (2018) [37]	Source : 36 outpatients (GA = 12, GB = 12, GC = 12) and mean age (S. D) = GA = 50 ± 6.33, GB = 58 ± 5.68, and GC = 55.75 ± 4.88	GA = MWM plus CPT GB = KT plus CPT GC = only CPT	Thrice per week for 2 weeks	VAS WOMAC	There was a statistically significant difference in each group and between groups Significant between-group differences were found	Both MWM and KT reduce pain and improve function, but there was a better improvement in group A

Kiran et al. (2018) [38]	Source : 62 outpatients (GA = 31, GB = 31) and mean age (S. D) : 47.47 ± 0.61	GA = MWM plus CPT GB = Maitland mobilization plus CPT	3 sessions per week for 2 weeks	VAS ROM WOMAC	The mean differences of both treatment interventions were significant	Patients in both groups showed improvement in pain, ROM, and functions

Bhagat et al. (2020) [39]	Source : 30 outpatients (GA = 15, GB = 15) and mean age (S. D) : GA = 53.73 ± 7.06, and GB = 56.87 ± 9.35	GA = MWM GB = Sham	3 sets with 10 repetitions, duration of follow-up not stated	NPRS TUG	NPRS and TUG are significantly improved in GA compared to GB after intervention	MWM was effective in improving pain and functional mobility in individuals with knee OA

Alkhawajah and Alshami (2019) [40]	Source : 40 outpatients (GA = 20, GB = 20) and mean age (S. D) : GA = 56.5 ± 7.6 and GB = 56.6 ± 8.5	GA = MWM GB = sham	3 sets with 10 repetitions for 2 days	VAS ROM WOMAC TUG	GA showed significant improvement in pain, TUG, and knee flexion ROM (*p* ≤ 0.026) But, WOMAC and knee extension ROM (*p*=0.067) were not significant	MWM was superior than sham in pain, physical function (walking), knee flexion and extension muscle strength, and knee flexion ROM for at least 2 days in patients with knee OA

Saddam Hussain Shaik et al. (2019) [41]	Source : 40 outpatients (GA = 20, GB = 20), mean age not mentioned	GA = MWM plus CPT GB = Maitland mobilization plus CPT	Three sessions per week for 6 weeks	KOOS Quadriceps peak torque	GA showed more statistical significance in improving pain and quadriceps peak torque than GB	MWM was more effective than Maitland mobilization

Pawar et al. (2019) [42]	Source : 20 outpatients (GA = 10, GB = 10), mean age not stated	GA = MWM GB = McConnell patella taping	Each session 15–20 minutes, four days a week	VAS	VAS is significantly improved in the experimental group (*P* < 0.0001) compared to the control group (*P*=0.0558)	MWM is comparatively more beneficial in reducing pain than taping in OA knee patients

Mahmooda et al. (2020) [43]	Source : 30 outpatients (GA = 15, GB = 15) and mean age (S. D) = 52.80 ± 6.32	GA = MWM plus usual care GB = Myofacial release plus usual care	Once a day, 5 days per week for two weeks	NPRS ROM WOMAC	Pain and ROM were improved in GA (*p* < 0.05). But, reduction of stiffness and improvement of physical function were seen in group B (*p* < 0.05)	MWM and myofacial release were effective for knee OA in pain, ROM, and functional abilities. However, MWM produced more quick outcomes than myofacial

Nigam et al. (2020) [44]	Soure : 40 outpatients (GA = 20, GB = 20) and mean age (S. D) : GA = 58.5 (4.36) and GB = 59.4 (6.57)	GA = MWM plus usual care GB = usual care	Three sets of 6–10 repetitions over two weeks at 6 months	WOMAC VAS ROM TUG	Significant effect of MWM in favor of GA for WOMAC and VAS was found. But, no significant difference between GA and GB was found for knee ROM and TUG	MWM provided clinically significant improvements in disability, pain, and functional activities six months later

Abbreviations: OA, osteoarthritis; MWM, Mulligan's movement with mobilization; MIMG, Macquarie injury management group; SWT, shock wave therapy; KT, Kinesio Taping; GA, group A (experimental group), GB; group B, GC; group C, CPT; conventional physical therapy, PJM; passive joint mobilization, VAS; Visual Analog Scale; WOMAC; Western Ontario and McMaster Universities Osteoarthritis Index, ROM; range of motion, NPRS; Numeric Pain Rating Scale, TUG; Time Up and Go, ALF; Aggregated Locomotor Function, KOOS; Knee injury and Osteoarthritis Outcome Scale.

**Table 3:** Risk-of-bias analysis.

Selection bias	Random sequence	Kandada and Heggannavar [32]
		Lalnunpuii et al. [5]
		Rao et al. [35]
		Kaya Mutlu et al. [36]
		Varma and Purohit [37]
		Bhagat et al. [39]
		Alkhawajah and Alshami [40]
		Mahmooda et al. [43]
		Nigam et al. [44]
	Allocation concealment	Lalnunpuii et al. [5]
		Rao et al. [35]
		Kaya Mutlu et al. [36]
		Bhagat et al. [39]
		Alkhawajah and Alshami [40]
		Nigam et al. [44]

Performance bias	Blinding of participants and treating therapist	Lalnunpuii et al. [5]
		Rao et al. [35]
		Kaya Mutlu et al. [36]
		Bhagat et al. [39]
		Alkhawajah and Alshami [40]
		Nigam et al. [44]

Detection bias	Blinding of assessor	Lalnunpuii et al. [5]
		Rao et al. [35]
		Kaya Mutlu et al. [36]
		Bhagat et al. [39]
		Alkhawajah and Alshami [40]
		Nigam et al. [44]

Attrition bias	Completeness of outcome data	Shenouda [31]
		Kandada and Heggannavar [32]
		Gupta and Heggannavar [33]
		Lalnunpuii et al. [5]
		Kulkarni and Kamat [34]
		Rao et al. [35]
		Kaya Mutlu et al. [36]
		Varma and Purohit [37]
		Kiran et al. [38]
		Bhagat et al. [39]
		Alkhawajah and Alshami [40]
		Saddam Hussain Shaik et al. [41]
		Pawar et al [42]
		Mahmooda et al. [43]
		Nigam et al. [44]
